# Capsular-Invasive Papillary Carcinoma in a Thyroglossal Duct Cyst With No Synchronous Thyroid Malignancy: A Management Dilemma

**DOI:** 10.7759/cureus.106140

**Published:** 2026-03-30

**Authors:** Virginia D Cuevas-Sandoval, Luis E Ocampo-Guzmán, Heberto Morales-Blake, Karla Biviano-Andrade, Juan Gómez-Rodríguez

**Affiliations:** 1 General Surgery, Hospital Regional Universitario de Colima, Colima, MEX

**Keywords:** open thyroidectomy, papillary carcinoma of the thyroid, sistrunk procedure, thyroglossal duct cyst malignancies, thyroglossal duct cysts

## Abstract

Thyroglossal duct cyst carcinoma is an uncommon entity, and the optimal management strategy continues to be debated, particularly regarding the indication for total thyroidectomy in addition to the Sistrunk procedure.

We report the case of a 38-year-old woman who presented with a painless midline neck mass. Imaging studies revealed a cystic suprahyoid lesion with a vascularized mural nodule containing calcifications, raising suspicion for malignancy. The patient underwent a Sistrunk procedure, and histopathological examination demonstrated a 1.5 cm intracystic papillary thyroid carcinoma with capsular invasion and negative surgical margins. Given these intermediate-risk features and abnormal scintigraphic findings, completion total thyroidectomy was performed; final pathology revealed no evidence of synchronous thyroid malignancy. The patient was referred for radioactive iodine therapy and remains under close follow-up without evidence of recurrence.

This case highlights the importance of maintaining a high index of suspicion when mural nodules are detected within a thyroglossal duct cyst and underscores the need for individualized management based on histopathological risk factors and thyroid imaging findings.

## Introduction

Thyroglossal duct cysts (TGDCs) represent the most common congenital midline neck anomaly, resulting from the incomplete involution of the thyroglossal tract during the embryologic descent of the thyroid gland [1]. Their estimated prevalence in the general population is approximately 7% [2,3]. Although most TGDCs are benign lesions, malignant transformation has been reported in a small proportion of cases, generally ranging from 0.7% to 1% [2].

Preoperative diagnosis is challenging because clinical presentation often mimics benign TGDC. Ultrasonographic features such as solid mural nodules and microcalcifications have been described as suspicious findings suggestive of malignancy [4,5]. However, fine-needle aspiration cytology demonstrates limited sensitivity due to the cystic nature of the lesion, with a reported diagnostic accuracy of around 60% [2,4].

Papillary thyroid carcinoma (PTC) is the predominant histologic subtype identified in TGDC-associated malignancies, accounting for more than 80% of reported cases. Other histologic variants, including follicular carcinoma and squamous cell carcinoma, have been described less frequently [2,6]. The pathogenesis remains controversial. Some authors support a de novo origin from ectopic thyroid tissue within the cyst wall, while others suggest the possibility of metastatic spread from an occult thyroid primary [2,7].

The Sistrunk procedure is considered the standard surgical treatment for TGDC. Nevertheless, the role of total thyroidectomy, neck dissection, and radioactive iodine therapy remains controversial, particularly in patients without clinical or radiologic evidence of thyroid involvement [1,2,7]. We present a case of papillary carcinoma arising within a TGDC with capsular invasion and discuss its management in light of current evidence.

## Case presentation

A 38-year-old woman with no significant medical history presented with a painless midline neck mass of three months' duration. She denied dysphagia, dysphonia, weight loss, or compressive symptoms.

Physical examination revealed a firm, well-circumscribed, midline suprathyroid mass measuring approximately 4 cm in greatest diameter. The lesion exhibited upward movement with both tongue protrusion and deglutition, consistent with the classical clinical features of a TGDC. No cervical lymphadenopathy was identified. Thyroid function tests were within normal limits.

**Figure 1:**
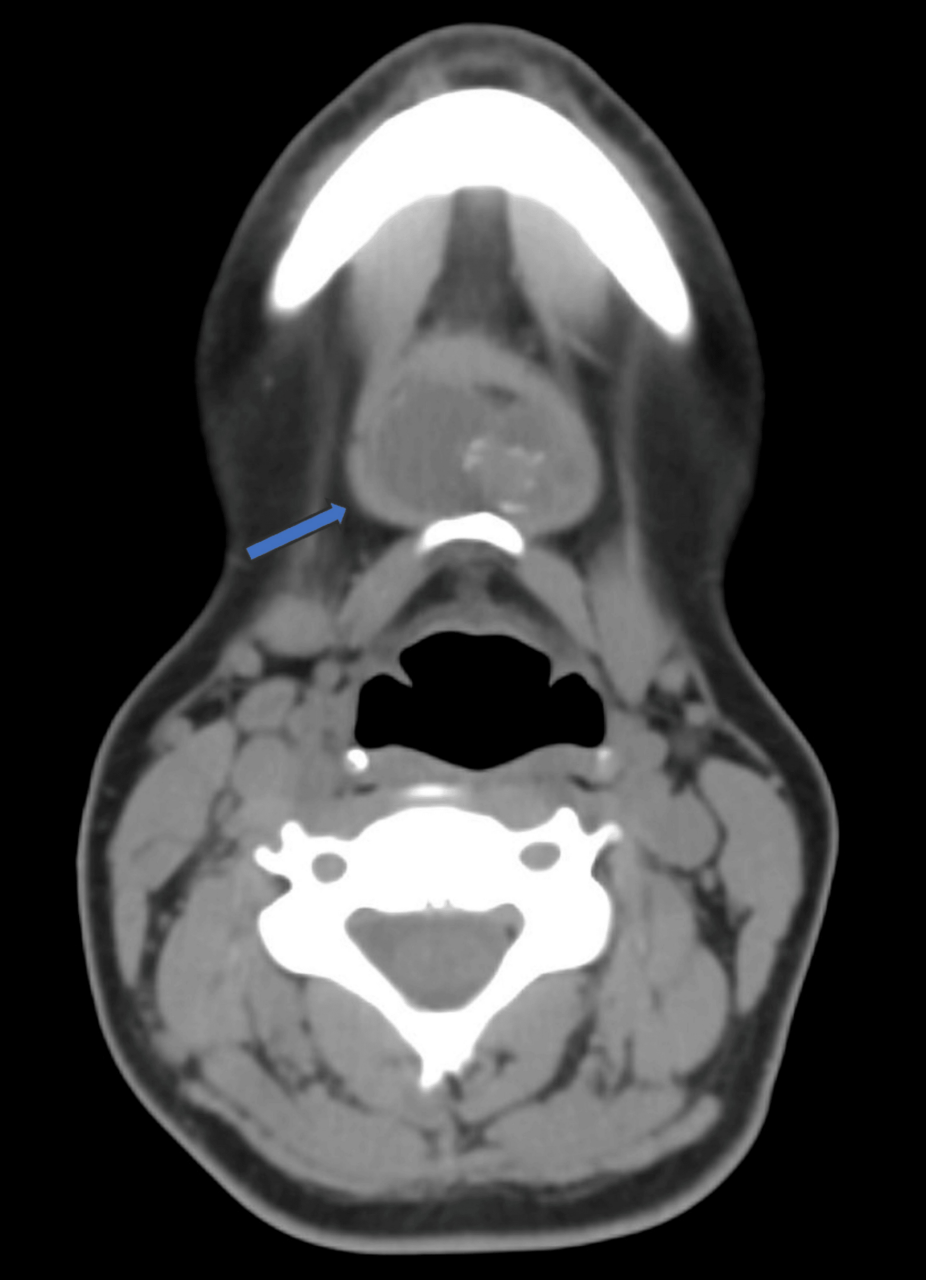
CT scan showing a cystic mass in the hyoid bone containing a solid component with calcifications CT: computed tomography

Neck ultrasonography demonstrated a heterogeneous hypoechoic cystic lesion with an internal vascularized mural nodule. The thyroid gland appeared normal in size and echotexture, without suspicious nodules or cervical lymphadenopathy. Contrast-enhanced computed tomography confirmed a suprahyoid cystic mass containing a solid mural component with punctate calcifications, raising suspicion for malignancy within a TGDC. The thyroid gland was visualized in its normal anatomical position, without radiologic evidence of focal lesions or pathological lymph nodes (Figure 1).

**Figure 2:**
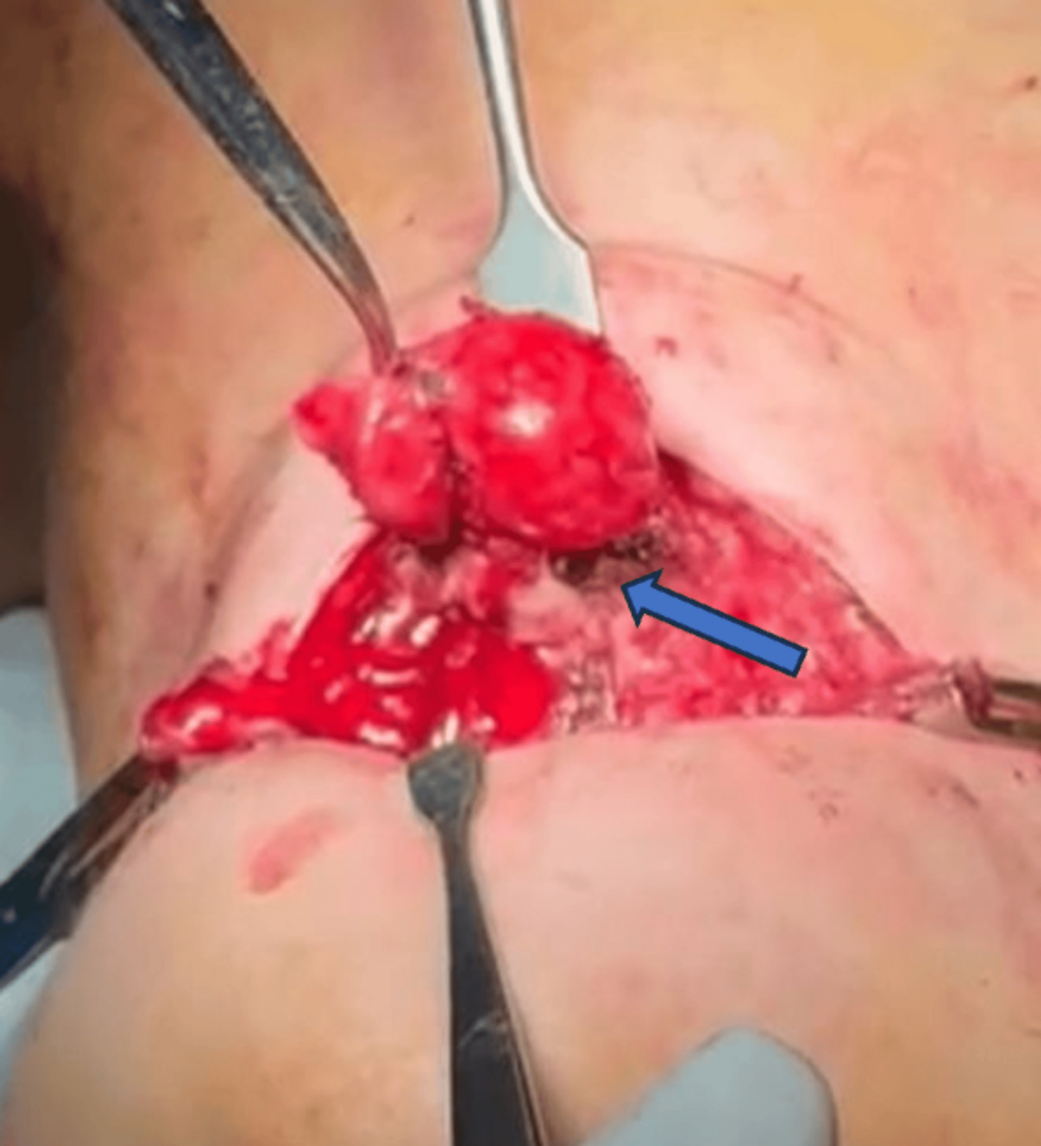
Sistrunk procedure showing a cystic lesion located above the hyoid bone

The patient underwent a Sistrunk procedure. Intraoperatively, a well-circumscribed cystic lesion located above the hyoid bone was identified and completely excised along with the central portion of the hyoid bone. There was no evidence of macroscopic adherence to the adjacent musculature or surrounding soft tissues, and no gross local invasion was observed (Figure 2).

**Figure 3:**
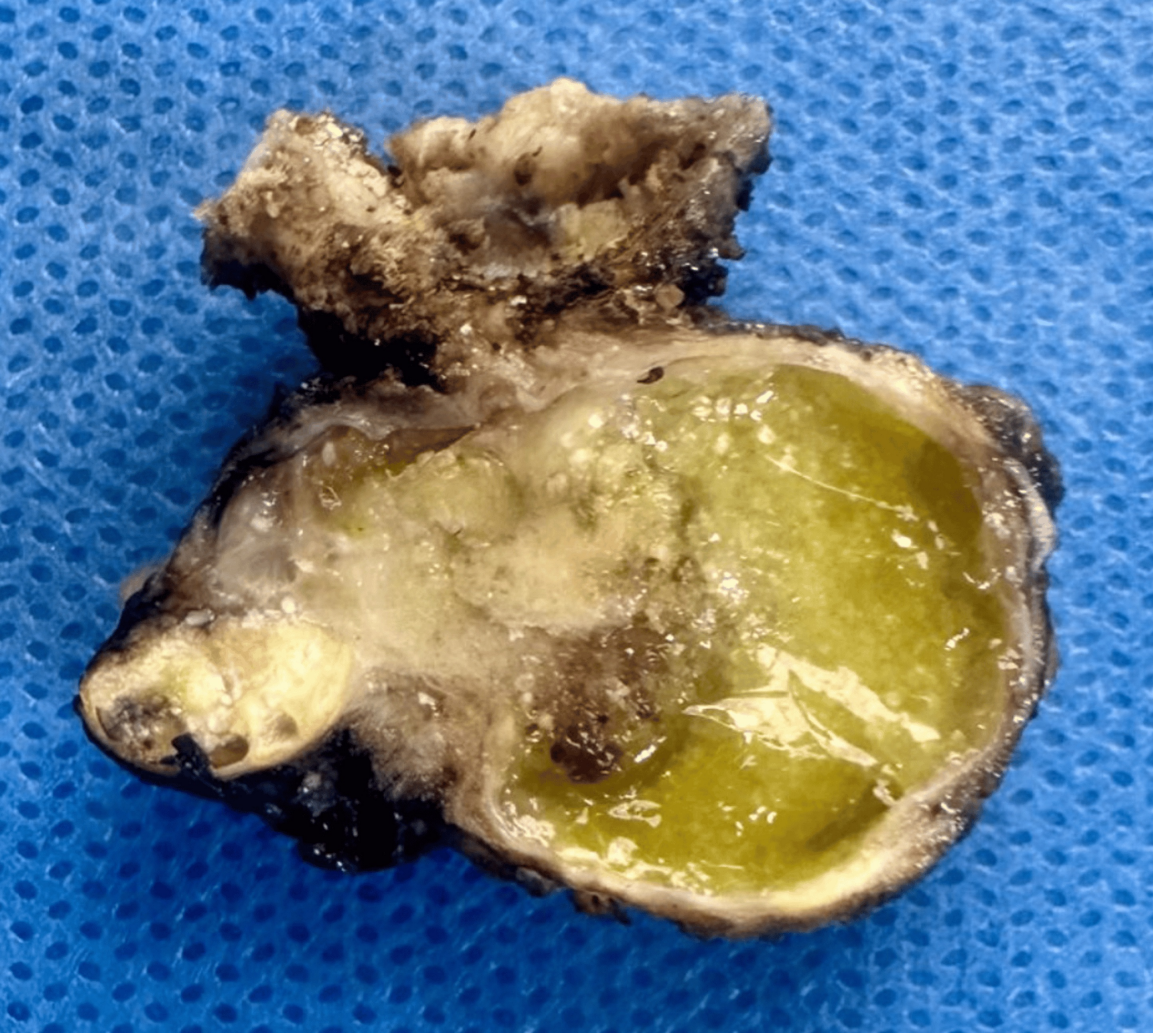
Histopathology revealing a 1.5 cm intracystic papillary thyroid carcinoma with capsular invasion

Histopathological examination revealed a 1.5 cm intracystic PTC with capsular invasion. No extracystic extension was identified. Surgical margins were negative, and no lymphovascular invasion was observed (Figure 3).

**Figure 4:**
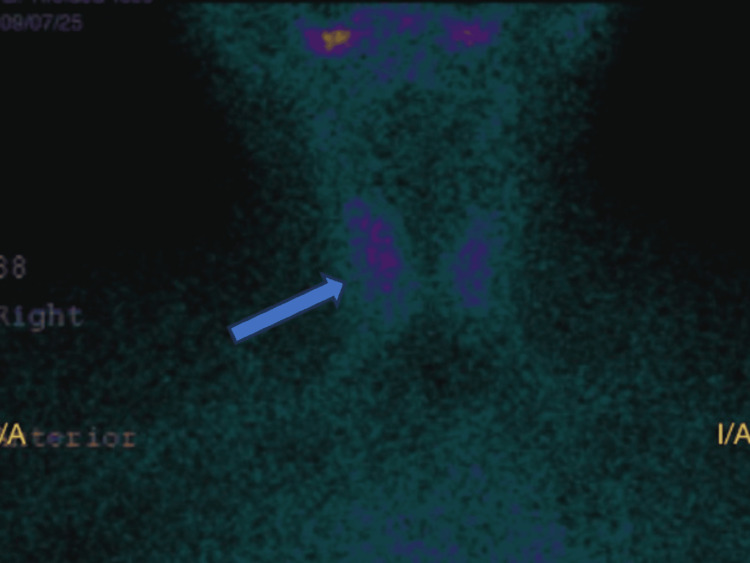
Thyroid scintigraphy demonstrating diffusely decreased uptake with focal cold areas in the inferior poles of both thyroid lobes (arrow), raising suspicion for possible occult thyroid pathology

Postoperative thyroid scintigraphy demonstrated diffusely decreased uptake in both thyroid lobes, with focal cold areas identified in the inferior poles of the left and right lobes (Figure 4). Although the primary tumor measured less than 2 cm, the presence of microscopic capsular invasion combined with these suspicious scintigraphic findings raised concern for possible synchronous thyroid pathology. Given these intermediate-risk features, completion total thyroidectomy was performed to exclude orthotopic thyroid malignancy, facilitate long-term surveillance with serum thyroglobulin levels, and allow the consideration of radioactive iodine therapy. Histopathological analysis of the thyroid gland showed no evidence of malignancy.

The patient was subsequently referred for radioactive iodine therapy and remains under endocrinological follow-up.

## Discussion

PTC arising within a TGDC is an uncommon but well-recognized clinical entity. The incidence of carcinoma in TGDC has traditionally been reported at approximately 1% [2,7], although institutional series demonstrate variability, with incidences ranging from 4% to over 6% in tertiary centers [3,6]. These findings suggest that malignancy may be more frequent than historically believed.

In our patient, malignancy was suspected preoperatively due to the presence of a vascularized mural nodule with calcifications on ultrasonography and computed tomography. This correlates with the findings described by Aculate et al., who emphasized that a central solid component within a cyst is a highly suggestive feature of malignancy [4]. The identification of these suspicious imaging characteristics allowed appropriate surgical planning.

Histologically, PTC accounts for the vast majority of TGDC-associated malignancies, representing over 90% of cases in large systematic reviews [2,7]. Our case demonstrated an intracystic papillary carcinoma with capsular invasion but negative margins and no lymphovascular involvement. 

The management of TGDC carcinoma remains controversial, particularly regarding the need for total thyroidectomy. Suresh et al. and Rayess et al. have reported significant rates of synchronous thyroid carcinoma, with Thimsen et al. documenting concomitant thyroid malignancy in 34.6% of cases undergoing thyroidectomy [1,2,7]. However, not all synchronous lesions impact survival, and completeness of the Sistrunk procedure has been shown to be the most important prognostic factor [7,8].

Risk-adapted management has therefore gained increasing support. Proposed high-risk factors include tumor size ≥2 cm, extracystic or capsular invasion, lymphovascular invasion, clinically evident lymphadenopathy, abnormal thyroid imaging, radiation exposure, and advanced age [2,7]. Paz-Ibarra et al. emphasized individualized decision-making in their case series, advocating tailored surgical strategies based on clinicopathologic findings [5].

In our case, the tumor measured 1.5 cm and demonstrated microscopic capsular invasion without lymphovascular involvement, extracystic extension, or nodal disease. Preoperative thyroid ultrasonography and computed tomography did not reveal suspicious nodules. However, thyroid scintigraphy performed after histopathological diagnosis and prior to completion surgery demonstrated diffusely decreased uptake with bilateral cold areas. Although postoperative changes may influence uptake patterns, the presence of these abnormalities raised concern for possible occult thyroid pathology. Following multidisciplinary discussion, completion thyroidectomy was performed as a risk-adapted strategy to definitively exclude synchronous malignancy and facilitate postoperative surveillance with serum thyroglobulin. Final thyroid histopathology was negative for carcinoma, underscoring the complexity and ongoing controversy surrounding the optimal extent of surgery in TGDC-associated PTC.

Prognosis in TGDC-associated PTC is excellent. Thimsen et al. reported overall survival rates exceeding 99% and recurrence rates below 10% [2]. Our patient remains under follow-up without evidence of recurrence, consistent with the favorable outcomes described in the literature [2,7,9,10].

## Conclusions

PTC arising within a TGDC is a rare but clinically relevant entity that should be suspected when mural nodules or other suspicious imaging features are identified. While the Sistrunk procedure remains the cornerstone of management, the role of total thyroidectomy continues to be debated and should not be considered mandatory in all cases. Rather, the extent of surgery should be guided by a comprehensive assessment of histopathological findings, thyroid imaging, and overall risk profile. This case highlights the complexity of surgical decision-making in TGDC-associated carcinoma and reinforces the importance of individualized, risk-adapted management and careful long-term follow-up.
